# Exploring the Diversity of Plant DNA Viruses and Their Satellites Using Vector-Enabled Metagenomics on Whiteflies

**DOI:** 10.1371/journal.pone.0019050

**Published:** 2011-04-22

**Authors:** Terry Fei Fan Ng, Siobain Duffy, Jane E. Polston, Elise Bixby, Gary E. Vallad, Mya Breitbart

**Affiliations:** 1 College of Marine Science, University of South Florida, Tampa, Florida, United States of America; 2 Department of Ecology, Evolution and Natural Resources, School of Environmental and Biological Sciences, Rutgers University, New Brunswick, New Jersey, United States of America; 3 Department of Plant Pathology, University of Florida, Gainesville, Florida, United States of America; 4 Gulf Coast Research and Education Center, University of Florida, Gainesville, Florida, United States of America; University of Kansas Medical Center, United States of America

## Abstract

Current knowledge of plant virus diversity is biased towards agents of visible and economically important diseases. Less is known about viruses that have not caused major diseases in crops, or viruses from native vegetation, which are a reservoir of biodiversity that can contribute to viral emergence. Discovery of these plant viruses is hindered by the traditional approach of sampling individual symptomatic plants. Since many damaging plant viruses are transmitted by insect vectors, we have developed “vector-enabled metagenomics” (VEM) to investigate the diversity of plant viruses. VEM involves sampling of insect vectors (in this case, whiteflies) from plants, followed by purification of viral particles and metagenomic sequencing. The VEM approach exploits the natural ability of highly mobile adult whiteflies to integrate viruses from many plants over time and space, and leverages the capability of metagenomics for discovering novel viruses. This study utilized VEM to describe the DNA viral community from whiteflies (*Bemisia tabaci*) collected from two important agricultural regions in Florida, USA. VEM successfully characterized the active and abundant viruses that produce disease symptoms in crops, as well as the less abundant viruses infecting adjacent native vegetation. PCR assays designed from the metagenomic sequences enabled the complete sequencing of four novel begomovirus genome components, as well as the first discovery of plant virus satellites in North America. One of the novel begomoviruses was subsequently identified in symptomatic *Chenopodium ambrosiodes* from the same field site, validating VEM as an effective method for proactive monitoring of plant viruses without *a priori* knowledge of the pathogens. This study demonstrates the power of VEM for describing the circulating viral community in a given region, which will enhance our understanding of plant viral diversity, and facilitate emerging plant virus surveillance and management of viral diseases.

## Introduction

Current knowledge of plant virus diversity is heavily biased towards agents of visible and economically important diseases, with less known about the potentially emergent viruses that have not yet made their presence known on crops. However, these undiscovered viruses can provide a reservoir of biodiversity for recombination and reassortment with existing pathogenic plant viruses, and are an essential component of the complex microbial ecology of plants [1], [2]. Much of this viral diversity may exist in plants without symptoms of disease; for instance, plants infected with viruses that are not yet adept at exploiting the new host, some of which have historically evolved into highly virulent pathogens [3], [4], [5].

While viruses and virus-like elements can be readily isolated from ∼60% of plants [1], even visibly diseased plants can sometimes have low viral titers [6], [7]. Additionally, not all infections result in the production of visible symptoms, and viruses can be limited to only certain tissues of the plant, such as actively growing tissues or the phloem [8]. Since the majority of known plant viruses are exclusively vector-transmitted [9], examination of insect vectors presents a unique avenue for exploring the diversity of plant viruses. The whitefly *Bemisia tabaci* species complex is an important vector of many plant viruses. Whiteflies often occur in high populations on many crops [10], [11], especially at the end of the production cycle when insecticide applications have been withheld due to harvest. The B biotype of *B. tabaci* feeds on a very wide range of plants [10], and is highly mobile, being able to fly short distances and capable of traveling up to several kilometers when assisted by the wind [12], [13]. In addition, begomoviruses can be retained for up to the lifespan of the adult whitefly [14]. Therefore, whitefly vectors are natural “flying syringes” that can sample viruses from many individual plants and different plant species over space and time.

Once the proper samples are obtained, an effective method of viral discovery is needed. Most viral identification techniques have limited capability for characterizing novel viruses due to their specificity. Common methods such as ELISA with antibodies for a specific virus, PCR or microarrays with primers designed for a specific virus, or PCR with degenerate primers that amplify a closely related group of viruses are effective methods for detecting close relatives of known viruses [15], [16], [17]. PCR has been used to characterize specific viruses in whiteflies and other insect vectors [18], [19], [20]; however, PCR-based methods do not allow for the discovery of novel viruses with low levels of similarity to previously described viral groups, and thus prevent a thorough exploration of viral biodiversity. The amplification of viral DNA by rolling circle amplification (RCA) followed by restriction digestion and cloning is a more recent approach that has facilitated begomovirus discovery from individual plants [21], [22]. However, this approach is restricted to circular ssDNA viruses, and the use of restriction analysis limits the types and numbers of viruses identified.

Viral metagenomics, a molecular technique that involves purifying viral particles from samples and shotgun sequencing the viral nucleic acids, circumvents the biases of classic viral identification methods, and has revolutionized the exploration of viral diversity [23], [24]. Viral metagenomics has been used to characterize viral communities present in the environment [25], [26], [27], as well as from individual plants [28] and animals [29]. The advantage of metagenomics for viral identification is that it allows for characterization of the complete viral community, including viruses with circular or linear genomes, and viruses that are too divergent to be detected by PCR assays based on known viral sequences. Purification of viral particles before sequencing ensures that the vast majority of the metagenomic sequences originate from viruses, in contrast to direct deep sequencing.

The vector-enabled metagenomics (VEM) approach presented here takes advantage of the highly polyphagous and mobile nature of the whitefly vector, combined with the capability of metagenomics to discover novel viruses without relying on sequence similarity to known viruses. This study utilized VEM to explore the diversity of DNA viruses in whiteflies collected from different crops in two agriculturally important sites in Florida.

## Results

### Summary of viromes

The VEM method, consisting of viral purification and metagenomic sequencing, was used to obtain sequence from DNA present inside virions in the whitefly vector, *Bemisia tabaci*. BLASTn analysis revealed that 79% of the sequences in the Citra virome and 93% of the sequences in the Homestead virome showed significant similarity to known plant viruses, with nucleotide identities between 70% and 100% to previously described begomoviruses (Table 1). Several sequences from the Homestead virome had short BLASTn alignments to the stem-loop and nonanucleotide sequence of begomoviruses, but further analysis of these sequences revealed that they belonged to novel satellite genomes. Each virome also had a small number of “unknown” sequences that did not have significant similarity to any available sequences in Genbank.

**Table 1:** Summary of BLASTn results for the virome sequences sharing nucleotide identities to the viruses in Genbank, showing the percent identity, and whether the reference viruses have been previously characterized in Florida.

BLASTn Identities to Known Viruses	>93%		88%–93%		<88%		Previously Characterized in Florida (Reference)
Component (DNA-A or DNA-B)	A	B	A	B	A	B	
**Closest Sequence in Genbank**							
**Citra**							
**Cucurbit leaf crumple virus**	**16** a	**18** a	**1** c	**7** c		**1** c	Yes ^[64]^
**Sida golden mosaic virus**		**2** a +					Yes *
**Tobacco leaf rugose virus**					**1** c #		No
**Homestead**							
**Anoda geminivirus**	**1** b						No
**Macroptilium golden mosaic virus**	**1** a		**4** c		**3** c ∧		Yes ^[65]^
**Malvastrum yellow mosaic Helshire virus**	**3** b		**7** c			**1** c	No
**Malvastrum yellow mosaic Jamaica virus**			**2** c			**1** c @	No
**Sida golden mosaic virus**	**5** a	**29** a	**2** c	**4** c	**2** c	**12** c	Yes *
**Sida golden yellow vein virus (monopartite)**	**9** b				**1** c		No
**Tomato yellow leaf curl virus (monopartite)**	**50** a		**10** c		**1** c		Yes ^[49]^
**Wissadula golden mosaic virus**			**1** c			**1** c	No

Sequences range from 100 to 700 nt in length, representing partial viral genomes. The whitefly viromes contain a diverse range of viral sequences, including

a sequences similar to viruses previously described in the area,

b sequences similar to viruses not previously identified in the area,

c Sequences that likely belong to new virus strains and virus species.

*Jones, L.A. direct GenBank submission.

+ Based on the full DNA-B sequence, this fragment belonged to a new strain of a novel.

Sida golden mosaic virus - [USA:Florida:Citra:2007:VEM] (SiGMV-[US:FL:Cit:07:VEM]).

# Based on the full DNA-A sequence, this fragment belonged to a new virus, tentatively named Chenopodium leaf curl virus - [USA:Florida:Citra: 2007:VEM] (ChLCV-[US:FL:Cit:07:VEM]).

∧ Based on the full DNA-A sequence, one of the fragments belonged to a strain of the recently identified Sida golden mosaic Linguanea virus, tentatively named SiGMLV—[US:FL:Hs:09:VEM].

@ Based on the full DNA-B sequence, this fragment belonged to a novel DNA-B sequence, tentatively named Whitefly VEM Begomovirus - [USA:Florida:Homestead:2009:VEM] (WfVEMBv-[US:FL:Hs:09:VEM]).

Further analysis of the viral sequences demonstrated that the two viromes contained sequences with a range of nucleotide identities to known begomoviruses (Table 1). According to Fauquet [30], entire begomovirus DNA-A components with >93% nucleotide identity are considered the same strain, DNA-A components with 88%–93% nucleotide identity are considered different strains of the same species, and DNA-A components with <88% nucleotide identity are considered new species. The results shown in Table 1 are based on analysis of individual metagenomic sequence reads ranging from 100 to 700 nt in length; the entire DNA-A would need to be sequenced before these viruses could be classified as novel strains and/or species.

### Citra whitefly virome

**Figure 1:**
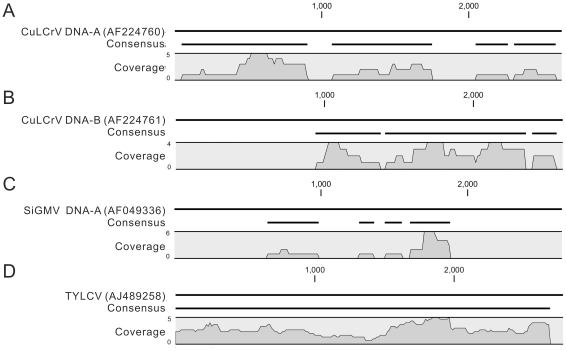
Coverage of known virus genomes obtained from the whitefly viromes. The reference genome from Genbank is shown in the top row of each panel, and the consensus represents the regions of the reference genome with coverage in the viromes. The numeric values represent the degree of genome coverage at each position. A & B) Near-complete coverage of Cucurbit leaf crumple virus DNA-A and DNA-B obtained from the Citra virome. C) Partial coverage of Sida golden mosaic virus DNA-A obtained from the Homestead virome. D) Complete coverage of the Tomato yellow leaf curl virus genome obtained from the Homestead virome.

The Citra whitefly virome was dominated by Cucurbit leaf crumple virus (CuLCrV), a begomovirus known to infect watermelon plants at this sampling site (Table 1). Alignment of the metagenomic sequences against a CuLCrV reference genome from Genbank demonstrated that VEM resulted in near-complete coverage of the DNA-A and partial coverage of the DNA-B (Figure 1A&B). The segments of both the DNA-A and DNA-B assembled from the Citra virome shared >97% nucleotide identities to the CuLCrV reference genome.

**Figure 2:**
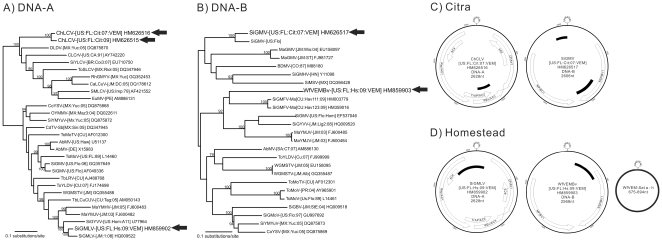
Genomic and phylogenetic analysis of the virus genomes discovered by VEM. The tree is midpoint rooted for clarity, all nodes with more than 75% bootstrap support are labeled, and new sequences are indicated by arrows. A) Maximum likelihood phylogeny of the entire DNA-A of the novel Chenopodium leaf curl virus - [USA:Florida:Citra: 2009], Chenopodium leaf curl virus - [USA:Florida:Citra: 2007:VEM] , and Sida golden mosaic Linguanea virus- [USA:Florida:Homestead:2009:VEM] with related begomovirus genome sequences. B) Maximum likelihood phylogeny of the entire DNA-B of the Sida golden mosaic virus - [USA:Florida:Citra:2007:VEM] and Whitefly VEM Begomovirus - [USA:Florida:Homestead:2009:VEM]. Begomovirus names are abbreviated according to convention. C & D) Genome characteristics and organization of the DNA-A, DNA-B and satellite DNA identified in the Citra and Homestead samples. The ORFs encode for: CP/AV1, coat protein; Rep/AC1, replication-associated protein; TrAP/AC2, transcription activator and/or silencing suppressor; REn/AC3, replication enhancer; MP/AV2, movement protein; AC4, AC4 protein; AC5, AC5 protein; MP/BC1, movement protein; NSP/BV1, nuclear shuttle protein. The positions of sequence fragments recovered in the virome, the conserved stem loop and nonanucleotide sequence are indicated.

Additionally, two sequences were identified that shared 96-97% nucleotide identities to the DNA-B of Sida golden mosaic virus (SiGMV). Based on one of these metagenomic sequences, PCR primers were designed to amplify, clone, and sequence the entire DNA-B from DNA extracted from the whiteflies. The resulting sequence had characteristics consistent with the DNA-B of SiGMV (Figure 2C), but shared only 90% nucleotide identity to Sida golden mosaic virus by whole component pairwise comparison. Therefore, this genome represents a divergent SiGMV DNA-B (Figure 2B), which we have named Sida golden mosaic virus - [USA:Florida:Citra:2007:VEM] (SiGMV-[US:FL:Cit:07:VEM]). It is likely that additional sequencing of this sample would also recover the corresponding DNA-A of this Sida golden mosaic virus.

Strikingly, one partial sequence of 243 nt was identified in the virome which had only 84% nucleotide identity to the DNA-A of Tobacco leaf rugose virus (Table 1). PCR primers were designed to amplify, clone, and completely sequence the DNA-A of this virus using DNA extracted from the whiteflies. The complete sequence of the DNA-A (2628 nt) of this virus only shared 81% nucleotide identity with its closest relative, a Mexican isolate of Desmodium leaf distortion virus (GenBank DQ875870)[31] (Figure 2A). Despite its low level of nucleotide identity to known begomoviruses, this viral sequence contains all the essential genome features of a begomovirus DNA-A (Figure 2C). The replication-associated protein, transcription activator, replication enhancer, and coat protein are present in an organization consistent with known begomoviruses, and the genome has a stem-loop containing the geminivirus-signature nonanucleotide sequence TAATATT/AC [32].

To investigate the host plant of this novel virus, a survey of 203 wild and cultivated plants was conducted at the Citra site where the whiteflies were originally collected. Degenerate PCR [15] for begomoviruses was positive in symptomatic *Chenopodium ambrosiodes*. A full length DNA-A clone (2626 nt) was obtained and sequenced from this plant, which was 96% identical to the complete DNA-A sequence identified from the whiteflies using VEM. This novel virus identified in *C. ambrosiodes* is tentatively named Chenopodium leaf curl virus - [USA:Florida:Citra: 2009] (ChLCV-[US:FL:Cit:09]), while the novel virus identified in the whiteflies through VEM is tentatively named Chenopodium leaf curl virus - [USA:Florida:Citra: 2007:VEM] (ChLCV-[US:FL:Cit:07:VEM]). Both viruses appear to be most closely related to Desmodium leaf distortion virus, but group with other New World begomoviruses such as Cotton leaf crumple virus, Sida yellow leaf curl virus and Tomato severe leaf curl virus (Figure 2A). Since none of these viruses have been previously reported in Florida, studies are underway to more fully characterize ChLCV.

### Homestead whitefly virome

For the Homestead sample, a number of metagenomic sequences had high (93–100%) nucleotide identities to known begomoviruses (Table 1). The virome was dominated by sequences from Tomato yellow leaf curl virus (TYLCV) and Sida golden mosaic virus (SiGMV) (Table 1). Complete coverage of the TYLCV genome was obtained, demonstrating that the TYLCV strain in these samples had a 29 base deletion compared to the reference genome (Figure 1D), which has also previously been detected in Texas, Arizona and Mexico [33]. Partial coverage of the SiGMV DNA-A was also obtained from the virome (Figure 1C).

Numerous metagenomic sequences shared between 88%–93% nucleotide identities to seven known begomoviruses (Table 1). Furthermore, 24 metagenomic sequences were found to have less than 88% nucleotide identities to these same seven begomoviruses (Table 1). PCR assays targeting several of these metagenomic sequences confirmed that they represented novel begomoviruses (see below), although it is important to note that the top BLASTn hit for the complete genome components was not always the same as the top BLASTn hit of the original metagenomic sequence.

PCR primers were designed to further explore two metagenomic sequences that initially showed low levels of identity to known begomoviruses. The complete DNA-A of one sequenced virus initially showed only 88% identity to its closest begomovirus relative; however, it is 94% identical to a Jamaican Sida virus that was deposited in GenBank in December 2010 (HQ009522, Figure 2A). We have tenatively named this virus Sida golden mosaic Linguanea virus [USA:Florida:Homestead:2009:VEM] (SiGMLV-[US:FL:Hs:09:VEM]). In addition, the complete DNA-B was sequenced of a virus, tentatively named Whitefly VEM Begomovirus - [USA:Florida:Homestead:2009:VEM] (WfVEMBv-[US:FL:Hs:09:VEM]), which shared only 80–81% nucleotide identity to its closest relatives, strains of Sida golden mosaic Florida virus isolated from malvaceous plants in Cuba (Figure 2B). The intergenic regions of SiGMLV-[US:FL:Hs:07:VEM] and WfVEMBv shared less than 90% nucleotide identity, thus they are likely to be genome components of two distinct viruses.

Eight metagenomic sequences were identified that had very short BLASTn similarities to the origin of replication and nonanucleotide sequence of begomoviruses, but otherwise shared no significant sequence identities to any known sequences. Using specific PCR assays, eight full circular genomes (675 to 694 nt) were cloned and sequenced. The fact that these genomes were present in virions in the whiteflies, have an adenosine-rich region, and do not have an open reading frame that codes for a replication-associated protein, suggests that these sequences are novel begomovirus satellites, which are tentatively named Whitefly VEM Satellite (WfVEM-Sat). The WfVEM-Sat genomes are similar to the Tomato leaf curl virus satellite (ToLCV-sat, Genbank U74627 [34], [35]) in size and genome organization. Pairwise comparisons among the eight satellite genomes ranged from 85–94% nucleotide identity (Table S1). Since all eight clones that were sequenced were unique, it is likely that continued sequencing would yield many more satellite genomes.

## Discussion

Using VEM, this study characterized whitefly-transmitted plant viruses circulating in whiteflies from two important agricultural regions in Florida. With the exception of Sida golden mosaic virus, the virus profiles of the two viromes were completely distinct from each other (Table 1). In addition to identifying pathogens that produce disease symptoms in crops, VEM enables the identification of viruses infecting native vegetation, newly emerging viruses that are not yet widespread in crops, and viruses that have more mutualistic interactions with their hosts [36]. The highly polyphagous and mobile nature of the B biotype of *B. tabaci* integrates viral diversity over space and time, making these insect vectors a perfect tool for sampling the viral community circulating in plants in a given region, including understudied weed hosts [37], [38], [39]. Performing viral metagenomics on whitefly vectors presents a unique approach to viral discovery that is complementary to existing plant viral discovery methods. Diseased plants sometimes have low titers of virions [6], [7] and infection can be limited to only certain tissues of the plant [8]. In comparison, viruses in whiteflies are likely to be present in virions and present in high titers [19], [40], making them ideal for the VEM approach which targets only intact (and thus potentially transmissible) viral particles. Metagenomic sequencing of viruses purified directly from whiteflies provides an additional advantage over traditional PCR-based approaches since it allows for the discovery of divergent viruses that might not be captured with existing degenerate PCR assays. A few recent studies have used metagenomic sequencing to examine RNA plant virus diversity [28], [41], [42]; however, to date, no published studies have applied metagenomic techniques to explore the diversity of plant DNA viruses. In addition, the approach of discovering viruses infecting plant or animal hosts by directly examining insect vectors that feed upon them is directly applicable to other host-vector systems (e.g., mosquitoes and human viruses; Ng et al., in review).

The viromes from both field sites have an extremely high percentage of identifiable viral sequences, with more than 79% of the sequences in each virome sharing nucleotide-level identities to known plant viruses. This is striking in comparison to DNA viruses from animals [43] or environmental samples [25], which are dominated by ‘unknown’ sequences with no significant homology to characterized genes. In addition, the sequences from environmental or animal viromes that have similarities to known viruses are typically only distantly related on the amino acid level, and rarely demonstrate nucleotide level identities. The fact that the majority of the whitefly virome sequences showed nucleotide level identities (BLASTn) to known plant viruses suggests that a more thorough understanding exists for whitefly-transmitted plant DNA virus diversity than for viruses infecting other hosts (e.g. animal viruses and bacteriophages). This finding also suggests that the majority of undiscovered DNA plant virus diversity consists of variations on viral themes that have already been described.

The vast majority of the sequences identified in this study were similar to begomoviruses. The single-stranded DNA (ssDNA) begomoviruses belonging to the family *Geminiviridae* are some of the most damaging and emergent viruses transmitted by whiteflies. Begomoviruses are often the limiting factor in the production of tomato, pepper, squash, melon, and cotton in the subtropics and tropics [44], [45], and periodic begomovirus epidemics in staple crops, such as cassava, have caused widespread famines in the developing world [46]. Some begomoviruses are associated with satellites, which can play a role in disease, and are dependent upon the begomovirus for replication and production of virions [47].

In this study, VEM led to the identification of multiple viruses from each site, exemplifying the advantages of this approach for describing the viral community circulating amongst plants in a given region, without *a priori* knowledge of what viral types are present. First, each virome was dominated by sequences with a high level of similarity to known viruses infecting the crop plants from which the whiteflies were collected. This demonstrates that VEM is capable of detecting and obtaining high levels of sequence coverage from active and abundant viruses in the primary collection host. Second, both viromes contained sequences similar to viruses that infect *Sida spp*., which are weeds commonly found near many agricultural fields in Florida. This demonstrates that discovery of viruses through VEM is not constrained by the collection host and that VEM is capable of identifying viruses from neighboring crops or weeds. Characterization of viruses from indigenous plants is especially interesting since this is a largely unexplored reservoir of genetic diversity that may contribute to emergence of novel begomovirus strains by host shift or recombination [38], [48]. Third, VEM is a powerful technique for discovering novel viruses, as evidenced by the recovery of sequences with either low levels of identity (<88%) or no significant similarity to previously described viruses. The discovery of novel satellite DNA sequences in this study is notable since these sequences are so divergent that they would not have been identified through degenerate PCR assays. Fourth, based only on single sequence fragments from the virome, the complete DNA-A or DNA-B of novel viruses, as well as numerous satellite genomes were obtained by PCR. This demonstrates that individual metagenomic sequences can be used as “hooks” to recover complete viral genomes. Finally, the novel virus ChLCV discovered in whiteflies was subsequently confirmed in a symptomatic weed collected from the study site two years later, successfully linking this sequence to a specific host plant and disease symptoms, and demonstrating that these viruses are persistent in the system.

This proof-of-concept study has helped expand our knowledge of begomoviruses in Florida. Vegetable crops in Florida have experienced significant losses due to whitefly-transmitted viruses over the past 15 years. The dominant viruses identified in this study were Tomato yellow leaf curl virus (TYLCV) and Cucurbit leaf crumple virus (CuLCrV), which are both whitefly-transmitted begomoviruses that have been introduced to Florida since the arrival of the silverleaf whitefly in the late 1980's [49], [50], [51]. CuLCrV is an emergent virus that infects cucurbits, which was not known in Florida until the virus impacted crop production in 2006 [51]. TYLCV was identified in Florida in 1997 [52] and has negatively impacted tomato production since that time. Identification of these two viruses provides an example of how VEM can be useful for early surveillance of emerging plant viruses. Sufficient depth of coverage for CuLCrV and TYLCV DNA-A was obtained such that had these viruses not already been described, the complete or near-complete genomes could have been assembled from the viromes (Figure 1). This is particularly noteworthy considering the small number of sequences obtained in this study. If the VEM method were performed using next-generation sequencing technologies, coverage of the dominant viruses would be much higher, and many more viruses present at lower abundances would be identified.

The small amount of sequencing performed in this study was sufficient to uncover numerous metagenomic sequence fragments related to viruses that had not been previously documented in Florida (Table 1). Some of these sequences are known to be present in neighboring Caribbean countries, and should now be monitored for in Florida, such as the recently identified Sida golden mosaic Linguanea virus from Jamaica. Furthermore, this study discovered numerous metagenomic sequences with <88% nucleotide identity to different genes of known begomoviruses (Table S2), which likely represent novel begomovirus strains or species. In fact, we have confirmed one of these novel begomoviruses by designing PCR assays to obtain its full component sequence (Figure 2), and found a host where it exists at high titer by subsequent field sampling. The metagenomic sequences produced in this study will enable future studies to characterize these new viruses' genomes and explore their origins, host ranges, and the timing of their introduction to Florida.

In addition to the discovery of begomoviruses, this study demonstrated the power of the VEM approach for the discovery of novel virus-associated entities such as satellites. This study is the first to demonstrate the presence of begomovirus satellites in the North America. Satellites can play important roles in plant disease, but often remain unrecognized due to limitations of common virus identification methods, especially when there is a lack of sequence homology with the helper virus. Most begomovirus satellites fall into one of several classes: α-satellites, β-satellites, and defective-interfering DNAs (DI-DNA) [2], [47]. All satellites are encapsidated by the helper virus coat protein, and share an origin of replication sequence, the conserved nonanucleotide sequence, with their helper virus. Beyond that short conserved sequence, α-satellites and β-satellites show no significant homology with their helper viruses, while DI-DNAs show extensive homology with their helper viruses. Until recently, both α-satellites and β-satellites were believed to be restricted to the Old World [2], [47], but the presence of several α-satellites have now been demonstrated in the New World [53], [54]. The satellites identified in this study are similar in size and organization to the Tomato leaf curl virus satellite (ToLCV-sat), which was first discovered in Australia in 1997 [34] and has been suggested to be a defective DNA β component [55]. Both WfVEM-Sat and ToLCV-Sat are distinct from α-satellites and β-satellites in terms of genome organization, lack of ORFs, and small genome size. The initial discovery of satellites through VEM will enable future studies to identify their helper virus(es) and associations with disease.

By enabling the discovery of a wide range of viruses and satellites, VEM is a powerful technique that will significantly enhance our fundamental scientific understanding of plant viral diversity, biogeography, and emergence. Typically, new plant viruses are not identified until an outbreak causing significant crop loss occurs. This has put agricultural biodefense into a reactive mindset – waiting until a new disease becomes a problem before trying to understand and combat the causative agent. The VEM approach circumvents that traditional process by obtaining a comprehensive sample of the viruses actively circulating in an insect vector population within a region, effectively integrating over individual plants, space, and time. Thus, VEM is a proactive molecular surveillance tool that allows rapid identification of emerging viruses before noticeable crop loss occurs, providing precious time for implementation of preventative measures and improving agricultural biosecurity.

Future studies using the VEM approach can incorporate high-throughput sequencing, expand the geographical range and frequency of sampling, and investigate both DNA and RNA viruses. The VEM approach can be extended to other insect vectors, such as aphids and leafhoppers, to understand the diversity of plant viruses that is being transmitted through each of these different vectors. In addition, further refinement of this method for begomovirus discovery should focus on recovering whole-genome components in order to allow for accurate analyses of recombination and evolution. When applied on a larger scale, VEM can describe global diversity of plant viruses, which will help refine existing plant virus phylogenies, aid in the development of more inclusive assays for monitoring introduced and emerging viruses, and increase our understanding of plant virus biogeography and evolution.

## Materials and Methods

### Sample Collection

Adult whiteflies (*Bemisia tabaci*) were collected using battery-operated vacuums [56] from two crop fields in Florida: Citra (29°24'N 82°06'W) and Homestead (25°28'N 80°30'W). The distance between the two collection sites was 460 km. The Citra sample contained whiteflies collected from soybean and watermelon plants in August 2007, while the Homestead sample was collected in April 2009 and contained whiteflies from tomato and squash plants in the vicinity of mixed cucurbit crops such as cantaloupe, pumpkin, cucumber, and watermelon. The two samples differed in terms of collection host, amount of sequencing performed, and the time and location of sampling. Upon collection, whiteflies were chilled at 4°C for 1 hr, plant debris and other insects were removed manually, and the whiteflies were stored at −80°C until processing.

### Virus Purification and Metagenomic Sequencing

Viruses were purified from the whiteflies using a modification of previously described methods [43], [57]. Briefly, the whiteflies were homogenized in sterile SM buffer (50 mM Tris, 10 mM MgSO_4_, 0.1 M NaCl, pH 7.5) and host cells were removed through centrifugation at 10,000*×g* for 10 minutes, followed by filtration of the supernatant through a 0.22 µm Sterivex filter (Millipore, Billerica, MA). The filtrate was treated with 0.2 volumes of chloroform for 10 minutes, then incubated with 2.5 U DNase I per µl sample for 3 hours at 37°C. DNA was extracted from the purified viral particles using the QIAamp MinElute Virus Spin Kit (Qiagen, Valencia CA) and amplified with the GenomiPhi V2 DNA Amplification Kit (GE Healthcare, Piscataway, NJ) according to the manufacturer's instructions. The GenomePlex Whole Genome Amplification Kit (Sigma-Aldrich, St. Louis, MO) was then used to fragment and amplify the viral DNA, which was subsequently cloned into the pCR4 vector using TOPO TA cloning (Invitrogen, Carlsbad, CA) and sequenced with the M13F forward primer by Beckman Coulter Genomics (Danvers, MA).

The resulting sequences were trimmed for vector and read quality using Sequencher 4.7 (Gene Codes, Ann Arbor, MI). A total of 58 and 158 sequences with >100 nt of good read quality after trimming were obtained from the Citra and Homestead viral metagenomes (viromes), respectively. All metagenomic data was deposited in Genbank under Accession numbers HN153414-HN153629. Metagenomic sequences were analyzed using BLASTn against the Genbank non-redundant database with an E-value cut-off of 1e-5 [58], [59]. Individual sequence reads were aligned to reference genomes from Genbank, and assembled into contiguous sequences (>95% identity over 30 nt) using Sequencher.

### PCR to complete genome components

To further characterize selected metagenomic sequences that likely represented novel virus species or strains, PCR primers were designed to amplify the entire DNA-A, DNA-B, and/or satellite DNA sequences (Table S3). The resulting whole genome PCR products were cloned and completely sequenced (Accession numbers HM626515-HM626517, HM859902-HM859911). The complete genomes were analyzed and annotated using Seqbuilder (DNASTAR, Madison, WI). Pairwise comparison of the genomes to reference genomes to determine percent identity was performed using BioEdit [60].

### Identifying the host for VEM generated viral sequences

To characterize the potential host of one of the novel virus sequences identified through the VEM approach, a survey of wild and cultivated plants was conducted at the Citra site in September and November 2009. Young leaves were collected from 203 plants belonging to 11 families and 15 genera and DNA was extracted using the Gentra Purgene Tissue Kit (Qiagen). Degenerate primers were used to screen for the presence of begomoviruses [15] and amplicons were sequenced and compared to the viromes by BLASTn. Once an amplicon sequence with high similarity to selected metagenomic sequences was identified, rolling circle amplification [22] was used to obtain a full length DNA-A clone from the original plant DNA extract, which was completely sequenced by primer walking.

### Phylogenetic Analysis

The completely sequenced genome components were aligned with their closest relatives from the GenBank non-redundant database using MUSCLE [61], and adjusted by eye in Se-Al (http://tree.bio.ed.ac.uk/software/seal/). Maximum likelihood phylogenetic trees were estimated with PAUP* 4.0 [62] using the general time reversible nucleotide substitution model specifying the proportion of invariant sites and a gamma distributed rate variation, which had been selected by MODELTEST [63], and tree-bisection-recombination branch-swapping. Bootstrap analyses (1000 replicate neighbor-joining trees) were used to assess the support for individual nodes on the phylogenetic trees.

## Supporting Information

Table S1 Pairwise comparison between the different Whitefly VEM Satellite DNA (WfVEM-Sat).
(DOC)

Table S2 Genes with similarity to the metagenomic sequences with <88% nucleotide identities to known begomoviruses. Sequences range from 100 to 700 nt in length, representing partial genome fragments.
(DOC)

Table S3 Primers used in this study.
(DOC)
