# Supplementary material for: Exploring the Diversity of Plant DNA Viruses and Their Satellites Using Vector-Enabled Metagenomics on Whiteflies
Source: PLoS One. 2011 Apr 22;6(4):e19050. doi: 10.1371/journal.pone.0019050 (PMC3081322; doi:10.1371/journal.pone.0019050)
Supplement: Table S1 — Pairwise comparison between the different Whitefly VEM Satellite DNA (WfVEM-Sat). (DOC) [file pone.0019050.s001.doc]

Table S1

|  | WfVEM-Sat a | WfVEM-Sat b | WfVEM-Sat c | WfVEM-Sat d | WfVEM-Sat e | WfVEM-Sat f | WfVEM-Sat g | WfVEM-Sat h |
| --- | --- | --- | --- | --- | --- | --- | --- | --- |
| WfVEM-Sat a | **100** |  |  |  |  |  |  |  |
| WfVEM-Sat b | **90.6** | **100** |  |  |  |  |  |  |
| WfVEM-Sat c | **92.9** | **89.0** | **100** |  |  |  |  |  |
| WfVEM-Sat d | **87.9** | **84.7** | **86.4** | **100** |  |  |  |  |
| WfVEM-Sat e | **94.0** | **89.7** | **92.0** | **89.5** | **100** |  |  |  |
| WfVEM-Sat f | **89.9** | **85.5** | **89.4** | **91.9** | **89.8** | **100** |  |  |
| WfVEM-Sat g | **85.9** | **88.1** | **84.9** | **89.6** | **85.2** | **89.2** | **100** |  |
| WfVEM-Sat h | **89.6** | **85.7** | **88.4** | **94.2** | **89.5** | **93.8** | **91.2** | **100** |
