# Supplementary material for: Exploring the Diversity of Plant DNA Viruses and Their Satellites Using Vector-Enabled Metagenomics on Whiteflies
Source: PLoS One. 2011 Apr 22;6(4):e19050. doi: 10.1371/journal.pone.0019050 (PMC3081322; doi:10.1371/journal.pone.0019050)
Supplement: Table S2 — Genes with similarity to the metagenomic sequences with <88% nucleotide identities to known begomoviruses. Sequences range from 100 to 700 nt in length, representing partial genome fragments. (DOC) [file pone.0019050.s002.doc]

Table S2

|  |  |  |  |  |  |  |  |  |
| --- | --- | --- | --- | --- | --- | --- | --- | --- |
| **Gene** | **AC1** | **AC2** | **AC3** | **AV1** | **AV2** | **BC1** | **BV1** | **Non-ORF** |
| **Citra** |  |  |  |  |  |  |  |  |
| ***Cucurbit leaf crumple virus*** |  |  |  | 1 |  |  |  |  |
| **Tobacco leaf rugose virus** |  |  | 1 |  |  |  |  |  |
| **Homestead** |  |  |  |  |  |  |  |  |
| ***Macroptilium golden mosaic geminivirus*** | 3 |  |  |  |  |  |  |  |
| ***Malvastrum yellow mosaic Helshire virus*** |  |  |  |  |  | 1 |  |  |
| ***Malvastrum yellow mosaic Jamaica virus*** |  |  |  |  |  | 1 |  |  |
| ***Sida golden mosaic virus*** |  |  |  |  |  | 1 | 11 | 2 |
| ***Sida golden yellow vein virus*** | 1 |  |  |  |  |  |  |  |
| ***Tomato yellow leaf curl virus*** | 1 |  |  |  |  |  |  |  |
| ***Wissadula golden mosaic virus*** |  |  |  |  |  |  | 1 |  |
