# Supplementary material for: Exploring the Diversity of Plant DNA Viruses and Their Satellites Using Vector-Enabled Metagenomics on Whiteflies
Source: PLoS One. 2011 Apr 22;6(4):e19050. doi: 10.1371/journal.pone.0019050 (PMC3081322; doi:10.1371/journal.pone.0019050)
Supplement: Table S3 — Primers used in this study. (DOC) [file pone.0019050.s003.doc]

Table S3

| **Genome** | **Primers 5' to 3'** |
| --- | --- |
| SiGMV-[US:FL:Cit:07:VEM] | GGATACGCTTATGGACGACG |
|  | TTCTCCACGGACAGTACACG |
| ChCLV-[US:FL:Cit:07:VEM] | TTCCTCCACCCTGTCTATCCT |
|  | GCTTCTGGGACGACGTACTT |
| SiGMLV-[US:FL:Hs:09:VEM] | TCCAATCATAGTGCGTCTGC |
|  | ACGATAGCGCGAGTGAAAAA |
| WfVEMBv-[US:FL:Hs:09:VEM] | TTCCACACTTGGTCAAATCG |
|  | ATTATTGTCTCGCGACGACG |
| WfVEM-Sat a & b | TCATTAGCTACCTCCTTTTGGC |
|  | TGAAGGTGGGGTTACTACGA |
| WfVEM-Sat c, d, & e | CAAGTGAGACACGCTGCAAA |
|  | GGCTATTGCAAATAATTAAGGC |
| WfVEM-Sat f, g, & h | CAGTTCTTTGCTTTGGCAGG |
|  | GGAGACCTAGCAAAACACCTC |
